# Dental caries risk indicators in early childhood and their association with caries polarization in adolescence: a cross-sectional study

**DOI:** 10.1186/s12903-016-0234-8

**Published:** 2016-07-02

**Authors:** Migle Zemaitiene, Ruta Grigalauskiene, Vilija Andruskeviciene, Zivile Kristina Matulaitiene, Jurate Zubiene, Julija Narbutaite, Egle Slabsinskiene

**Affiliations:** Department of Preventive and Pediatric Dentistry, Medical Academy, Lithuanian University of Health Sciences, Luksos- Daumanto 6, LT-50106 Kaunas, Lithuania

**Keywords:** Dental caries risk indicators, Early childhood, Caries polarization, Adolescence

## Abstract

**Background:**

Based on the hypothesis that biological and social risks accumulate during life, it is important to identify possible dental caries risk indicators from the life course of early childhood and assess their association with caries polarization in adolescence.

**Methods:**

A cross-sectional design was applied to the study, and a multistage cluster sampling method used to draw a representative sample of 1063 18-year-old Lithuanian adolescents. The dental examinations were performed according to the methodology for oral status evaluation recommended by the World Health Organization. Parents of the participating adolescents completed a self-administered questionnaire about their children’s life course during early childhood. The interdependence of characteristics was evaluated by chi-square (*χ*^2^) and Student’s (t) criteria. A multivariate logistic regression model with the Significant Caries (SiC) index as an outcome was performed.

**Results:**

The mean scores for the number of decayed, missing, and filled teeth (DMFT) and decayed teeth (DT) in the SiC positive group were higher than the corresponding values in the SiC negative group (6.14 [SD, 2.30] and 1.67 [SD, 2.02] vs 1.28 [SD, 1.11] and 0.34 [SD, 0.69], *p* < 0.001, respectively). Three dental caries risk indicators were identified that were independently associated with a SiC positive outcome: gender(OR = 1.32 [95 % CI: 1.01–1.73]), earlier eruption of the first primary tooth(OR = 1.43 [95 % CI: 1.03–1.97]), and past caries experience in the primary dentition (OR = 1.62 [95 % CI:1.22–2.14]).

**Conclusions:**

These study findings provide reliable evidence that gender, earlier eruption of the first primary tooth, and past caries experience in the primary dentition should be considered to be dental caries risk indicators and may have an adverse effect on caries polarization in adolescence.

## Background

Dental caries is one of the most prevalent chronic diseases, affecting most children, adolescents and adults worldwide [1]. It is induced by biological and environmental factors. Factors related to dental caries are researched in studies to provide more information about individuals who have high caries risk, and the identification of these factors makes it easier to select the most important groups to target in preventive dentistry programs.

Factors that have been associated with dental caries in cross-sectional studies are considered to be risk indicators, while factors detected in longitudinal studies are considered to be risk factors [2]. It was found that 106 dental caries risk indicators are associated with caries development in children [3], including indicators from the period of early childhood. Caries risk predictors are factors related to the caries process, but are currently not thought to be etiological; the validity of these factors is also determined in longitudinal studies [4]. According to many studies, past dental caries experience is the main predictor of future dental caries experience irrespective of additional predictors [5–8]. However, biological, behavioral, and social indicators of the early childhood life course, such as developmental characteristics at birth, general socioeconomic background, and upbringing features, could also contribute to future dental caries experience.

Non-homogeneous distribution of dental caries in some populations shows that polarization of dental caries exists [9]. The Significant Caries (SiC) index has been proposed to bring attention to the individuals who have the highest caries values in every population; it is a mean DMFT of the one third of the study population with a highest caries score [10]. Dental caries polarization is usually related to socioeconomic deprivation [11]. Thus, socioeconomic factors have also been identified as risk indicators for dental caries development [12]. In this study, the SiC index was chosen to assess which caries risk indicators are associated with the one third of individuals with the highest DMFT scores in a studied population.

Whereas it is hypothesized that accumulation of the biological and social experiences from the early childhood life course may increase the likelihood of future dental caries, the aim of the present study was to evaluate the role of dental caries risk indicators and self-reported oral health in early childhood and their association with caries polarization among adolescents in Lithuania.

## Methods

### Study design and population

This was a cross-sectional study based on caries examination and parental interviews using a structured questionnaire. It was conducted in 2014 among 18-year-old Lithuanian adolescents and their parents. A multistage cluster sampling method was used to draw a representative sample of 1063 adolescents and their parents. Lithuania is divided into 10 counties. In the first stage, each county was divided into smaller urban and rural administrative units (clusters). During the second stage, in each cluster, the first and last schools (sub-clusters) on the alphabetic list of all the schools were selected (based on data from the education management information system of the Centre of Information Technologies in Education). In the third stage, gymnasium class (a block) was selected. One hundred parents of chosen adolescents from each selected block were asked to complete the questionnaire. In total, 2000 adolescents from across the country were approached. Only 1063 of these met the inclusion criteria (age range 17.5–18.5 years and voluntary agreement to participate in this study). The sample size was calculated using Paniott’s formula with an error of 0.05 % based on the 18-year-old population; this amounted to 37,036 according to Statistics Lithuania. Using this formula, it was determined that at least 396 18-year-old adolescents should be included in the study.

### Caries examination

The dental examinations were performed according to the methodology of oral status evaluation recommended by the World Health Organization [13], under standardized conditions using the dental chairs available in the school’s dental offices and portable dental units equipped with a halogen light source, compressed air and a suction device. The examinations were performed by two pediatric dentists who were trained and calibrated to record the parameters of oral health. Training and calibration was performed on 35 18-year-old subjects who were not included in the final sample. Cohen’s kappa statistic was used to test the reliability of observations made by two different examiners (inter-examiner) and two observations of each examiner made at two different points of time (intra-examiner) at the baseline and after 1 year, at the end of the study. Reliability of the caries diagnostic criteria was assessed at the tooth surface level. The kappa value for inter-examiner reliability was 0.92, and for intra-examiner reliability the values ranged from 0.92 to 0.94.

To identify the group of adolescents in which dental caries was polarized, the SiC index was used [14]. Individuals who had the highest third of DMFT scores formed the SiC positive group, while all others formed the SiC negative group.

### Data collection

Participating parents completed an anonymous self-administrated questionnaire of 25 questions. They were asked about their children’s early childhood, including information about biological factors: the mother’s pregnancy course, preterm birth, and child birth weight. They were also asked to provide information about when their child’s first deciduous and permanent teeth erupted, at what age the child first visited the dentist, and whether the child had experienced caries in the deciduous teeth. Social determinants, such as the mother’s education level and the child’s birth order, were also included in the survey. Pilot study of the questionnaire was performed 6 months’ prior the study. The questionnaire was additionally administrated for a 50 participants who were not included in the final sample. Corrections to the questionnaire were made according to the comments presented by the parents.

To determine the extent to which all the questions in the questionnaire measured the same concept, Cronbach’s alpha coefficient was calculated. Measurement of internal consistency gave a coefficient of 0.8, which showed a high level of reliability for the questionnaire. Validation of the questionnaire was performed through the assessment of content validity. Content validity was examined in order to assess the extent to which a questionnaire measures what it is intended to measure.

### Statistical analysis

Statistical data analysis was performed using SPSS 22.0 (Statistical Package for the Social Sciences for Windows). In accordance with the principles of descriptive data analysis, the mean of the quantitative variables with a standard deviation (SD) was presented. The interdependence of qualitative characteristics was evaluated with the chi-square (*χ*^2^) criterion. If variable distribution met the distribution normality assumption, Student’s (t) criterion was applied to compare the quantitative size of two independent groups. When the variables did not meet the distribution normality condition, a significance level was verified by a nonparametric method using the Mann–Whitney test.

Receiving operating characteristic (ROC) curve analysis helped to decide where to draw the line between sensitivity and specificity. It was used to assess the accuracy of predictions and determined the optimal cut-off value.

The probability of an event given a certain risk indicator was calculated using logistic regression analysis, including odds ratio (OR) and its confidence interval (95 % CI). For complex evaluation of probability, the multivariate logistic regression model was used. The threshold for statistical significance was set at *P* < 0.05.

Cohen’s kappa statistic and Cronbach’s alpha coefficient were calculated to check the reliability of the dental caries examination and questionnaire, respectively.

## Results

A total of 1063 18-year-old adolescents (427 males and 636 females) were enrolled in the study. The overall 18-year-old Lithuanian adolescent population had a mean DMFT score of 2.93 (SD, 2.81), as reported by the authors previously [15].

Characteristics of the studied population including dental caries risk indicators, using the SiC index as an outcome, are shown in Table 1. Mean DMFT (6.14 [SD, 2.30]) and mean DT (1.67 [SD, 2.02]) scores in the SiC positive group were higher than the mean DMFT (1.28 [SD, 1.11]) and mean DT (0.34 [SD, 0.69]) scores in the SiC negative group (*p* < 0,001). Statistically significant differences were observed between males and females in the SiC positive group (36 vs 64) and the SiC negative group (43 % vs 57 %). Dental caries indicators such as early eruption of the first primary tooth at a mean age of 6.94 (SD, 2.38) months, and past caries experience in primary teeth (55 % of individuals were affected), had a bigger impact on individuals in the SiC-positive group, and a statistical difference was recorded. However, no difference was observed between developmental characteristics at birth, the child’s first dental visit, mother’s education level, and birth order.

**Table 1:** Characteristics of 18-year-old Lithuanians in relation to SiC negative and SiC positive outcomes

Characteristic	SiC negative *N* = 701	SiC positive *N* = 362	P	OR [95 % CI]
Age (%)				
18 years	66 %	34 %		
Gender (%)				
Male	296 (43 %)	131 (36 %)	*P* = 0.040	1.32 [1.01–1.73]
Female	405 (57 %)	231 (64 %)		
Residence (%)				
Urban area	491 (70 %)	243 (67 %)	*P* = 0.330	
Rural area	210 (30 %)	119 (33 %)		
DMFT [SD]	1.28 [1.11]	6.14 [2.30]	*P* < 0.001	
DT [SD]	0.34 [0.69]	1.67 [2.02]	*P* < 0.001	
FT [SD]	0.89 [0.99]	4.14 [2.51]	*P* < 0.001	
Normal pregnancy course?				
Yes	93 %	93 %	*P* = 0.893	
Child born prematurely?				
Yes	75 %	77 %	*P* = 0.716	
Child’s birth weight (kg)	3.45 [0.55]	3.51 [0.49]	*P* = 0.137	
Child’s birth order?				
First	56 %	50 %	*P* = 0.102	
Second or >	44 %	50 %		
Age at eruption of first primary tooth? (months)	7.18 [2.23]	6.94 [2.38]	*P* = 0.046	1.43 [1.03–1.97]
Age at eruption of first permanent tooth? (years)	6.29 [1.19]	6.09 [0.95]	*P* = 0.090	
Age at first visit to dentist? (years)	5.78 [2.37]	5.66 [2.37]	*P* = 0.236	
Primary teeth affected by caries? (past caries experience)				
Yes	43 %	55 %	*P* < 0.001	1.62[1.22–2.14]
Mother’s education level?				
Did not finish high school	7 %	9 %	*P* = 0.447	
Completed high school	27 %	27 %		
Higher non-university education	31 %	33 %		
Higher university education	35 %	31 %		

The first primary tooth erupting at 7.5 months of age was the optimal cut-off value determined by ROC curve analysis. The SiC positive group consisted of 251 individuals (69) whose first primary tooth eruption was earlier than 7.5 months of age and 111 individuals (31 %) whose first eruption was later than 7.5 months of age (*P* = 0.033).

The ratio of chance for the individual to be assigned to the SiC positive group was higher if the individual was female (OR = 1.32 [95 % CI: 1.01–1.73]), the first primary tooth erupted earlier (OR = 1.43 [95 % CI: 1.03–1.97]), or if the primary teeth were affected by dental caries (OR = 1.62 [95 % CI: 1.22–2.14]). These three risk indicators were independently associated with SiC positive outcome (Table 1).

The results of the multivariate logistic regression analysis are presented in Table 2. It shows the complex evaluation of the probability of being assigned to the SiC positive group. 66 % of individuals were correctly predicted by the model.

**Table 2:** Results of multivariate logistic regression analysis predicting the probability of a SiC positive outcome

Dental caries risk indicators	OR [95 % CI]
Female	1.36 [0.98–1.88]
Past caries experience	1.87 [1.37–2.55]
First primary tooth erupted earlier than 7.5 months of age	1.38 [0.99–1.94]
Constant = −**1.54**; *P* < 0.001	

*OR* odds ratio, *CI* confidence interval

## Discussion

The period of early childhood spans from birth to 8 years [16]. During this time, many factors related to dental caries affect children. Some may have an impact on the individual’s future life and oral health. Maternal pregnancy disorders such as intrauterine growth restriction or preeclamsia are associated with increased risk of preterm delivery or low birthweight [17, 18]. Preterm and low birthweight children are more susceptible to dental caries [19]. This is related to an increased incidence of enamel hypoplasia, which encourages earlier and more prolific colonization of *Streptococcus mutans,* which generally translates into a higher caries rate [20]. It has been suggested that adolescents born extremely preterm are at higher risk of poorer oral health, including dental caries [21]. However, in the present study, no association was found between these dental caries risk indicators and caries polarization.

Early eruption of teeth has been proposed as a dental caries risk indicator on account of the fact that the teeth are exposed to the cariogenic environment for a longer period. It has been found that early childhood caries can develop in primary teeth as early as 10–12 months of age [22]. These findings are consistent with the findings of our study, which showed that eruption of the first primary tooth earlier than 7.5 months increases the risk of higher DMFT scores in adolescence. However, no coherent relationship was determined between early eruption of the first permanent tooth and dental caries polarization. This was surprising because earlier permanent teeth eruption is more characteristic of females than males, because of females’ earlier onset of maturation [23], and in the studied population we found a strong association between gender and caries polarization: females recorded higher DMFT scores. The gender of participants was only relevant in relation to the eruption of permanent teeth, because no significant difference was found between males and females in the eruption of primary teeth [24]. Females tend to be at a higher risk of dental caries for many reasons: hormonal fluctuations during puberty, different saliva composition and flow rate, social role in the family [25]. All these factors could make the oral environment more cariogenic for females than for males.

Past caries experience in the primary dentition seems to be a fundamental risk indicator for dental caries. In longitudinal studies this indicator is called a dental caries predictor. It was found that past caries experience in the primary dentition is a more valid predictor of present caries in the permanent dentition than caries experience in the first permanent molars [26]. The findings of our current study are consistent with other studies that found that past caries experience has an impact on caries polarization [27]. Among 4–6-year-old Lithuanians, caries prevalence ranges between 79 and 97 % [28]. One potential reason for this is the lateness of children’s first visit to the dentist. According to our data, the mean age for the child’s first visit to the dentist was 6 years, which is far from the recommended age of 1 year [29]. This finding might explain the high caries prevalence among Lithuanian preschool children, but it was not associated with caries polarization in adolescence.

Socioeconomic risk indicators such as the mother’s education level are associated with poorer knowledge about oral health and hygiene. A higher education level could help an individual to acquire a better job and a higher income, and thus guarantee a better socioeconomic position [30], including easier access to dental services and oral hygiene products [31]. Socioeconomic indicators are mostly related to the incidence of dental caries in small population groups; however, neither the mother’s education nor the subject’s birth order were associated with caries polarization in the present study. Additionally, the subjects’ area of residence (urban or rural) was not significantly associated with caries polarization.

This study is unique in its concept, given that there is a lack of studies in which adolescents’ parents are interviewed about their children’s early life course. There is a theory that biological and social risks accumulate during life, and that the most susceptible period is early childhood, during which such factors as low birthweight, periods of illness, adverse environmental conditions and behaviors compound, and the risk of chronic diseases, including dental caries, increases in later life [32].

The some limitations should be considered when interpretenting the findings of our study. The cross-sectional design of the study allows us to discuss only dental caries risk indicators, leaving risk factors and risk predictors for future longitudinal studies. We also admit that parents may not precisely remember detailed events of their children childhood especially if they have more than one child. The reliability of the memories is quite difficult to assess objectively, therefore we presume that this issue did not have an impact on results, although participants were informed about the importance of accurate responses. In addition, the response rate in present study reached 53 %. One more possible minor limitation of this study could be the potential bias associated with the questionnaire data as well.

## Conclusions

Experience of disadvantage in early childhood could have a deleterious effect on adolescents’ oral health. Our findings provide reliable evidence that female gender, early eruption of the first primary tooth, and past caries experience in the primary dentition may be dental caries risk indicators and increase the risk of dental caries in adolescence. These indicators are related to caries polarization, and identification of these indicators could help to draw the attention of policy makers and practitioners to the individuals who are at a higher risk of dental caries.

## Abbreviations

CI, confidence interval; DMFT, decayed missing, and filled permanent teeth; DT, decayed permanent teeth; OR, odds ratio; ROC, Receiving operating characteristic; SD, standard deviation; SiC, Significant Caries Index; SPSS, Statistical Package for the Social Sciences for Windows; *χ*^2^, chi-square criterion; *t:* Student’s criterion
